# The contrasting role of male relatedness in different mechanisms of sexual selection in red junglefowl

**DOI:** 10.1111/evo.13145

**Published:** 2017-01-05

**Authors:** Cedric Kai Wei Tan, Philippa Doyle, Emma Bagshaw, David S. Richardson, Stuart Wigby, Tommaso Pizzari

**Affiliations:** ^1^ Department of ZoologyEdward Grey Institute, University of OxfordOxfordOX1 3PSUnited Kingdom; ^2^ School of Biological SciencesUniversity of East AngliaNorwichNR4 7TJUnited Kingdom

**Keywords:** Ejaculate expenditure, *Gallus*, kin recognition, kin selection, rare male effect, sperm competition

## Abstract

In structured populations, competition for reproductive opportunities should be relaxed among related males. The few tests of this prediction often neglect the fact that sexual selection acts through multiple mechanisms, both before and after mating. We performed experiments to study the role of within‐group male relatedness across pre‐ and postcopulatory mechanisms of sexual selection in social groups of red junglefowl, *Gallus gallus*, in which two related males and one unrelated male competed over females unrelated to all the males. We confirm theoretical expectations that, after controlling for male social status, competition over mating was reduced among related males. However, this effect was contrasted by other sexual selection mechanisms. First, females biased male mating in favor of the unrelated male, and might also favor his inseminations after mating. Second, males invested more—rather than fewer—sperm in postcopulatory competition with relatives. A number of factors may contribute to explain this counterintuitive pattern of sperm allocation, including trade‐offs between male investment in pre‐ versus postcopulatory competition, differences in the relative relatedness of pre‐ versus postcopulatory competitors, and female bias in sperm utilization in response to male relatedness. Collectively, these results reveal that within‐group male relatedness may have contrasting effects in different mechanisms of sexual selection.

Competition among males over mating is often intense, and traits that confer a competitive advantage are favored by sexual selection (Darwin 1871; Andersson 1994; Shuster and Wade 2003). Four distinct mechanisms of selection are recognized to operate on males at successive stages of the reproductive process: male competition and female preference determine differential mating success, and, when females mate with multiple males and their ejaculates overlap, both intra‐ and intersexual selection can continue after mating through: sperm competition (Parker 1970), and cryptic female choice (Eberhard 1996), respectively.

Theory predicts that, as in other forms of competition, male investment in intrasexual competition should be modulated by population structure. This prediction often emerges as property of inclusive fitness arguments: when local competitors are more genetically related to each other than to the average individual in the population (i.e., they are “positively” related), reduced competition for access to mating opportunities may yield indirect fitness benefits (e.g., Kokko and Lindström 1996; Boomsma 2007; Wild et al. 2011; Pizzari and Gardner 2012; Díaz‐Muñoz et al. 2014; Faria et al. 2015; Pizzari et al. 2015). A similar logic has been used to predict that males should invest less in ejaculates competing with the ejaculates of related competitors after mating (Parker 2000).

The role of within‐group male relatedness in sexual selection is, however, potentially complex. First, the effect of relatedness on competition can change due to population structure, the proximate mechanisms of competition, or the scale of competition (Pizzari et al. 2015). For example, Taylor (1992) demonstrated that, owing to local competition, indirect benefits derived by helping relatives (or competing less intensely with them) can be counterbalanced by direct costs such as fewer mating opportunities. In other words, when competitors are all similarly related to each other (either because they are all unrelated or all closely related), the relative relatedness between an actor and a recipient is effectively zero (i.e., not divergent from the population average), removing indirect benefits associated with preferentially helping kin. On the other hand, when relatedness varies, and an actor is able to preferentially benefit individuals that are sufficiently more closely related to itself than the population average (i.e., positively related), indirect benefits may promote the evolution of cooperation among relatives. Second, nonrandom interactions may also relax competition and favor cooperation among unrelated competitors, for example through reciprocity, manipulation, or public goods (e.g., Temeles 1994; Clutton‐Brock 2002; Patzelt et al. 2014; Díaz‐Muñoz et al. 2014; Pizzari et al. 2015).

Empirical investigations of the role of within‐group male relatedness in sexual selection have largely focused on precopulatory male competition, for example lekking birds and mammalian male coalitions (Díaz‐Muñoz et al. 2014), while fewer have considered postcopulatory competition (Pizzari et al. 2015). This narrow focus, however, tends to ignore the role played by females, which can bias the mating success of a male (precopulatory female choice) or the fertilization success of his ejaculates (postcopulatory cryptic female choice). Specifically, relatedness among males may inform female decisions, and these female‐driven biases need to be considered when assessing the role of male relatedness in sexual selection. For example, females may prefer to mate with (or favor the sperm of) males that are genetically different from each other to increase the genetic diversity of their offspring (Jennions and Petrie 2007). Alternatively, females might prefer males that are genetically related to each other because of the immunological costs of mating with genetically diverse mates. A recent study of *Drosophila melanogaster* found that females preferred to remate with novel males that were related to their first partners (i.e., “genetically familiar”; Tan et al. 2013).

Similarly, current empirical approaches also often neglect the role of male relatedness in male investment in sperm competition (Pizzari et al. 2015), e.g. in terms of sperm allocation (Parker and Pizzari 2010). Yet, there may be important differences in patterns of male competition before and after mating. For example, precopulatory competition is often mediated by costly fights, which may have lasting repercussions for a male, while after mating males compete through their ejaculates. In other words, when a male loses a fight to mate with a female (i.e., precopulatory competition), this will not only reduce his reproductive success in the current reproductive event but the injuries associated with this fight may also have longer lasting consequences, for example by hampering his reproductive success in future reproductive events and even his survival. In contrast, losing sperm competition will reduce the reproductive success of a male in the current reproductive event but is unlikely to bear similarly long‐term consequences. In addition, dispersal, female sperm storage capacity, and mechanisms of sperm competition can all result in drastic differences in the scale of pre‐ versus postcopulatory male competition. In species where males compete locally to mate and females move to different patches to remate, male competition over mating tends to occur locally while sperm competition ensuing from female remating and sperm storage will occur on a more global scale (Wild et al. 2011; McDonald et al. 2013; McDonald and Pizzari 2014). On the other hand, if males share partners with similarly related males, the ejaculates locked in sperm competition within a female might be equally related to each other, removing scope for cooperation (Pizzari and Foster 2008). However, the way in which successive inseminations change the relative relatedness of sperm within a female is also likely to depend on patterns of sperm displacement, sperm precedence, and female sperm storage (Parker and Pizzari 2010). Mechanisms of cryptic female choice (Greeff and Parker 2000; Ball and Parker 2003) and trade‐offs in male investment in pre‐ versus postcopulatory competition are also expected to influence strategies of male sperm allocation (Parker et al. 2013).

In this study, we experimentally investigate the role of within‐group male relatedness across multiple mechanisms of sexual selection: precopulatory male–male competition and female mate choice, and postcopulatory sperm competition and cryptic female choice, in a captive population of red junglefowl, *Gallus gallus*.

Populations of red junglefowl are structured in small social groups (Collias et al. 1966; Collias and Collias 1967; Sullivan 1991; Collias and Collias 1996), where male social status governs male access to mating opportunities through its role in male–male competition (Leonard and Zanette 1998; Johnsen et al. 2001). In small social units, socially dominant males tend to mate with more females, and have preferential access to these females: they mate repeatedly with their partners and interrupt copulation attempts by other males (Collet et al. 2012). Part of this advantage is mediated by female behavior: females often display a marked preference to mate with socially dominant males (Leonard and Zanette 1998; see Wood‐Gush 1971; Pizzari 2001 for similar patterns in domestic fowl, *G. domesticus*). However, females are also typically polyandrous as a result of both a female propensity to seek copulations from multiple males (Ligon and Zwartjes 1995; see Wood‐Gush 1971 for similar patterns in domestic fowl), and male sexual coercion (Collet et al. 2014; see Pizzari and Birkhead 2000 for feral domestic fowl). Polyandry, in combination with female ability to store viable sperm for prolonged periods of time (Parker et al. 1942; Etches 1996; Pizzari et al. 2008), creates opportunity for postcopulatory mechanisms of sexual selection: sperm competition and cryptic female choice. Studies of domestic fowl show that relative sperm numbers play a key role in determining the outcome of sperm competition (Taneja and Gowe 1962; Martin et al. 1974; Etches 1996), particularly so for ovulations occurring shortly following insemination (Pizzari et al. 2008). Male fowl plastically adjust the number of sperm allocated to individual copulations in response to aspects of the socio‐sexual environment, including phenotypic cues of female fecundity, female sexual novelty, and the perceived level of sperm competition (Pizzari et al. 2003). Patterns of differential sperm utilization by females consistent with cryptic female choice have also been detected in junglefowl and feral populations of domestic fowl. Females appear to bias sperm retention against inseminations by socially subordinate males (Thornhill in Birkhead and Møller 1992; Pizzari and Birkhead 2000; Dean et al. 2011), and by related partners (Pizzari et al. 2004; Løvlie et al. 2013). Importantly, this latter response indicates the possibility that fowl may be able to recognize kin. This is quite plausible given that kin recognition has already been demonstrated in other phylogenetically close species of galliformes (Bateson 1982; Waldman and Bateson 1989; Petrie et al. 1999). Kin recognition may be especially relevant in a species like the red junglefowl, where individuals exhibit limited dispersal and social groups comprise members of varying degrees of relatedness, which often leads to sexual interactions among closely related individuals (Collias and Collias 1996). This population structure may therefore lead to males often competing with relatives over reproductive opportunities.

We studied small social units in which three males competed for access to females all genetically unrelated to the males, and where two males were genetically related to each other but unrelated to the third male. This approach, based on variable relatedness between males, enabled us to test the specific prediction arising from current theory that a focal male should invest less in competition with a relative than with an unrelated male, both before and after copulation. We also sought to test the hypothesis that females may respond differentially to within‐group male relatedness, by favoring males either related or unrelated to other competitors (but always unrelated to the female). For example, a female propensity to increase the genetic diversity of her brood would occur through a bias in favor of the male unrelated to the other two males, a pattern, which in this context, may be explained by a preference for rare male types (O'Donald 1977; Partridge 1988). Because such bias may occur before and/or after copulation, we investigated both patterns of female behavior in precopulatory female choice, and patterns of female sperm utilization in postcopulatory cryptic female choice, after controlling for male sperm allocation. We first conducted an experiment to study precopulatory dynamics, that is male competition and female mate choice; we then conducted two experiments to quantify the role of male relatedness on postcopulatory sexual selection, that is sperm competition (measured in terms of male sperm allocation), and cryptic female choice.

## Material and Methods

### STUDY POPULATION

The study was conducted on a population of red junglefowl, *G. gallus* at the Oxford University Field Station in Wytham, Oxfordshire, over three breeding seasons, August–September 2010, May–June 2011 and May–June 2012. Individuals were genotyped at between 16 and 26 (median = 16) variable microsatellite loci out of those detailed in Table S1. Male relatedness was measured using a coefficient of relationship (*r*), calculated based on pairwise similarity of individual microsatellite genotypes (Queller and Goodnight 1989). Throughout the study, two males were considered “related” if 0.45 < *r* <0.6, and “unrelated” if –0.05 < *r* <0.05; and females were always unrelated to one another and to all males in a trial (*r* < 0.05). In an attempt to decouple relatedness from social familiarity, all birds utilized in the study were artificially hatched and raised in batches comprising multiple sib groups. Prior to the start of each experiment, birds were randomly assigned to individual trials and males were kept isolated from the females for at least two days to allow replenishment of sperm reserves, while females were kept isolated from males for at least two weeks to ensure depletion of sperm reserves from previous matings (Parker et al. 1942).

### PRECOPULATORY EXPERIMENT

**Figure 1:**
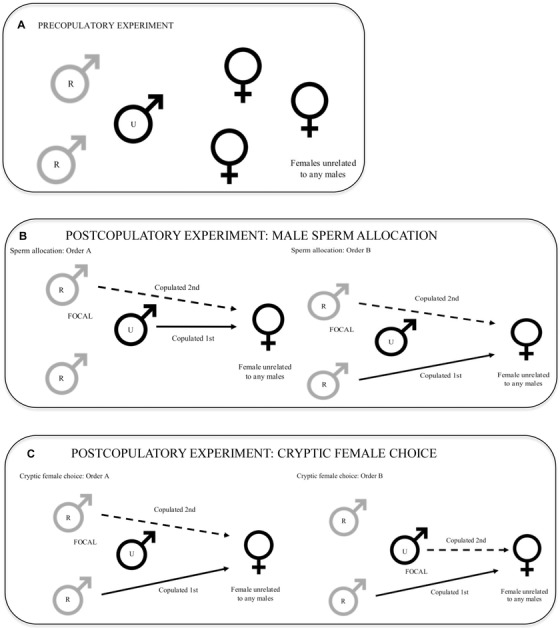
Experimental design. R–related male type; U–unrelated male type. (A) Precopulatory trials. (B) Postcopulatory experiment investigating sperm allocation. Males are the same in both orders. (C) Postcopulatory experiment investigating cryptic female choice. The focal male is the same male for both orders, the other males are different in different orders.

We studied precopulatory behavior in groups of three males and three females in outdoor pens (2010: n_trials_ = 12, n_males_ = 21, n_females_ = 18; 2011: n_trials_ = 16, n_males_ = 25, n_females_ = 14). This group size was within the natural range of size and sex ratio observed in natural groups (Collias and Collias 1996). In each group, two of the males were related while the third was unrelated to either of the related males. All females were unrelated to one another and to the males (Fig. 1A). The three males were assembled in a group on day 0 and the social hierarchy of the group was established by monitoring the outcome of dyadic interactions throughout this and the following day (i.e., day 1), following an established protocol (Froman et al. 2002). Briefly, a male was considered the loser in any interaction if he retreated one body length or more from the approaching rival male (Johnsen et al. 2001; Froman et al. 2002; Wilson et al. 2009). Male A was considered dominant over male B when B avoided A in the majority of encounters (Guhl et al. 1945; Froman et al. 2002). This enabled us to assess male status in the absence of females and thus independently of reproductive opportunities. We could then utilize social status to predict the level of aggression displayed by a male toward another male over specific mating opportunities when females were subsequently introduced to the group.

Males were exposed to females on three separate 2‐hour observation periods: on the afternoon (4–6 pm) of day 1, and on the morning (7–9 am) and afternoon (4–6 pm) of day 2. After each observation, females were removed from the pen and sexually isolated from the males until the next observation. Sexual behavior has marked temporal patterns in this species. First, there are two daily peaks: in the morning and late afternoon (Pizzari and Birkhead 2001). Second, female propensity to mate tends to decline as females accumulate matings over successive days (Løvlie et al. 2005). This experimental design enabled us to focus on peaks of sexual behavior (i.e., morning and evening in the first 2 days of female exposure to males). Crucially, by removing females at the end of each of observation period, we ensured that no unobserved matings influenced patterns of sexual behavior, for example by increasing female resistance to further mating (Løvlie et al. 2005) or by increasing the sexual familiarity (Pizzari et al. 2003). To remove the possibility of changes in the male hierarchy, we continued to monitor male status throughout the trial (day 1 and 2). We found that male hierarchy on day 0 strongly predicted the male hierarchy during the experimental trial (Fig. S1).

We sought to conduct behavioral observations blind with respect to within‐group male relatedness, by using naïve observers whenever this was possible. Specifically, CT planned the grouping while PD, EB, and CT conducted the observations and experiments. Neither PD nor EB knew the relatedness of the males and CT collected data without explicit recall or referral to the male relatedness information. We tested for an observer effect in the most important behavioral responses (see below) and found no evidence of this (Proportion of mating attempts interrupted: Observer ID: *X* ^2^_2_ = 0.190, *P* = 0.909; Proportion of mating attempts resisted by females: Observer ID: *X* ^2^_2_ = 1.860, *P* = 0.395; Average female resistance: Observer ID: *X* ^2^_2_ = 1.517, *P* = 0.468).

#### Male–male competition

In each observation period, male and female identity was recorded in all copulation attempts. We monitored: (i) any case when a male attempted to interrupt the copulation attempt of another male, both in 2010 and 2011. In 2011, we also recorded: (ii) the level of aggression displayed by the interrupter in 2011, which was scored on a gradient of 1–3 (1: male tried to interfere with no physical contact with attempting male; 2: male interrupted with physical contact; 3: male interrupted mating attempt and attacked the attempting male), and (iii) the number of aggressive events (chasing, “waltzing,” fighting) between two males (Rushen 1984). Finally, we recorded the number of male‐initiated attempts in 2010 and 2011, and, in 2011, the number of courtship events performed by individual males before mating attempts, to examine the effect of male type (related or unrelated) on male behavior toward females. The level of aggression displayed by individual males was consistent across the three observation periods (i.e., day 1 pm, day 2 am, and pm; Fig. S2).

#### Female preference

Female response to different male types (related or unrelated; R or U) was measured as the: (i) level of female resistance (1–5; 5 being the highest level of resistance; Løvlie and Pizzari 2007), (ii) proportion of copulation attempts resisted (4–5 on the resistance score), and (iii) probability of solicitation to a male (1 on the resistance score). Copulation was recorded as successful if the male lowered his train over the female's cloaca or if there was cloaca contact.

### POSTCOPULATORY EXPERIMENTS

#### Sperm allocation to sperm competition

This experiment examined a focal male's sperm investment to a female in response to his relatedness with a male competitor. Three males (two related and one unrelated to each other) were housed together one day before each trial to enable the establishment of a dominance hierarchy. One of the two related males was randomly chosen as the focal male. On day 1, one of the nonfocal males (related or unrelated to the focal male) was allowed to copulate with a female unrelated to all males, in full view of the other males. Immediately after this copulation, the focal male was presented with the same female and allowed to copulate with her (Fig. 1B order A). After a minimum of two days when his sperm reserves were replenished, the focal male was allowed to copulate with the same female after the other type of male (related or unrelated) copulated with her (Fig. 1B order B). In other words, if the first nonfocal male was the unrelated male type, the second nonfocal male was the related male type. This design enabled us to interpret variation in the number of sperm allocated during a trial, controlling for the confounding effect of sperm depletion generated by previous matings. To avoid potential order effects, the order with which the nonfocal related male or nonfocal unrelated male was allowed to copulate with the female was alternated in a balanced design.

Unlike the precopulatory experiments, where females were of the same age as the males (4 years old in 2010), females used in the postcopulatory trials were either 4 years old or 1 year old because of a limited number of available females. Females were fitted with a harness covering their cloaca, allowing ejaculate collection following an established protocol (Pizzari et al. 2003; Pizzari 2007). Males may sometimes copulate without releasing any sperm (“aspermic copulation,” Løvlie et al. 2005); therefore in each trial, a male was given up to 20 minutes to produce one spermic copulation (Pizzari et al. 2003). As an indication of the focal male's propensity to copulate, we recorded the time elapsed to first spermic copulation, and collected the resulting ejaculate. Ejaculate volume was measured to the nearest 0.5 μL using a Gilson pipette. To quantify the density of sperm, 2.5 μL of the ejaculate was mixed with 197.5 μL of phosphate buffer saline solution and light absorbance measured at 595 nm wavelength with a spectrophotometer (Scientific Laboratory Supplies, UV 1101). If the semen sample was too diluted or concentrated, we added more semen or phosphate buffer saline solution respectively, and thereafter adjusted the absorbance value accordingly. The number of sperm in the ejaculate was then calculated through a standard curve as a function of light absorbance (Ciereszko and Dabrowski 1993; Donoghue et al. 1996). We conducted a total of 31 paired trials (n_focal males_ = 17, n_nonfocal males_ = 27, n_females_ = 22) in 2011.

#### Cryptic female choice

This experiment examined the possibility that females may bias sperm utilization after mating in response to within‐group male relatedness. Three males (two related and one unrelated to each other) were housed together prior to each trial. In each group, a focal male was randomly assigned as one of the related males or the unrelated male. On day 1, a female unrelated to any of the males of the group was fitted with a harness and presented face‐to‐face to one of the nonfocal related male of the group, in an adjacent experimental pen, in full view of the males of the group. Thereafter, the female was turned around and mounted by this male. Immediately after this copulation the first male was returned to his group, and the focal male (the other related male type or the unrelated male type) was presented with the same female and allowed to copulate with her without the harness (Fig. 1C order A). We recorded the copulation using two Toshiba Camileo X400 camcorders placed at a right angle relative to each other and focusing on the female cloaca. Female ejection or acceptance of an ejaculate was determined following an established protocol (Dean et al. 2011). Briefly, ejection was detected by direct observation of the mating pair and confirmed through analysis of video recording (see Dean et al. 2011). Videos were scored by CT, and confirmed by an experienced researcher (Rebecca Dean) who was blind to the relatedness treatments. Out of 44 cases (22 paired trials), one insemination failed because the ejaculate was misplaced by the male, and for six additional cases video analysis did not resolve the outcome of the insemination. These seven trials were excluded from further analysis. In four cases, part of the ejaculate was misplaced by the male but the rest of the ejaculate was inseminated successfully, and no ejection was observed in these four cases. When ejection occurred, we collected and quantified the amount of semen that was ejected (Dean et al. 2011). We used a 200 μL pipette to measure the volume of semen to the nearest 1 μL and a spectrophotometer to measure the absorbance value. These values were used to estimate the number of sperm in the sample using a standard curve (Bakst and Cecil 1997).

Male groups were reassembled and the focal male was placed with two other males to reverse his relatedness type (e.g., if the focal male was the unrelated type in the previous group, he would be housed with a relative and an unrelated male to change his type to related in the new group; Fig. 1C order B). The focal male was given a minimum of 48 h from the previous trial to allow complete replenishment of his extragonadal sperm reserves (Etches 1996). The experimental protocol was then repeated with the focal male allowed to copulate with another female shortly after she was mounted by one of the related male type, as outlined above. To avoid potential order effects, the order with which the focal male was the related or unrelated male type was alternated in a balanced design. This design enabled us to compare female postcopulatory responses to the ejaculate of the same focal male when he played the related and unrelated type.

After insemination, females were kept in pairs and fed with colored lipid dyes (Sudan black or Sudan red, Daddi 1896) to assign the maternity of individual eggs. Eggs were collected for the following 10 days, opened and identified as belonging to either female using the color of the yolk. We then quantified the number of sperm reaching individual eggs as the number of sperm‐induced hydrolysis points on the outer perivitelline layer (PVL) of the egg (Pizzari et al. 2004), which is a sensitive measure of probability of fertilization by an ejaculate (Wishart 2006). Variation in the number of sperm‐induced hydrolysis points, after controlling for confounding factors such as the volume of semen inseminated and the time elapsed from insemination, provides scope to detect female‐driven postcopulatory biases in sperm utilization. While this approach is useful to reveal potential female biases, it is not designed to test how such biases affect paternity share when multiple males inseminate the same female and sperm competition occurs. We conducted a total of 22 paired trials (n_focal males_ = 12, n_nonfocal males_ = 14, n_females_ = 16) in 2012.

### STATISTICAL ANALYSIS

#### Male–male competition

All analyses were conducted using R 3.0.2. To investigate the way within‐group male relatedness modulates the intensity of male–male competition, we analyzed the effect of male relatedness on male–male aggression displayed over mating events, using generalized linear‐mixed models (GLMM) in the lme4 package in R (Bates and Maechler 2009). Two separate GLMMs analyzed variation in two male response variables: “proportion of mating attempts interrupted” with Binomial error distribution, “number of aggressive interactions” with Poisson error distribution. “Aggression level of interruption” was analyzed in a cumulative link mixed model (ordinal package; Christensen 2015). For the analysis on “proportion of mating attempts interrupted,” each mating attempt by a male was represented twice in the dataset, to record whether either of the other two males interrupted the attempt (coded as yes or no). This allows for the analysis of the proportion of mating attempts that were interrupted by each male type, accounting for four possible unrelated interactions (two either related male interrupts the unrelated male, RU, plus two the unrelated male interrupts either related male, UR) versus two possible related interactions (one related interrupts the other and vice versa). For the analysis on “number of aggressive interactions,” each pair of males was represented twice in the dataset, representing both directions where a male could be the aggressor or a recipient. Therefore, for each trial, there were six rows and if there were no aggressive interactions, we placed the response value “0.” This again adjusted for the four possible unrelated interactions (RU, UR) versus two possible related interactions (RR). We then followed up these analyses breaking down the RU, UR, and RR categories of interactions. Values reported in figures and tables are average per male values.

The propensity of a male to interrupt the copulation attempt of another male and the level of aggression displayed in such interaction are likely to be predicted to a degree, by the social status of these males (Pizzari 2001). There is however considerable residual variation in male–male aggressiveness that is independent of status (McDonald and Pizzari, unpubl. data). Our analyses therefore asked whether—after controlling for social status—the relatedness between two males influenced the per male probability of copulation disruption and the level of aggression displayed over mating opportunities. In all models, “male relatedness” (related or unrelated) and “relative dominance” (dominance of interrupting male vs dominance of attempting male; H = higher, L = lower) were entered as fixed factors, “relatedness:relative dominance” was entered as an interaction, and “Year” (1 or 2) as a fixed factor if the data were collected over two years. The variable “relative dominance” is not sensitive to small differences in the hierarchy (e.g., it assumes that the difference between an interrupting male of status 1 and an interrupted male of status 3 is similar to the difference between an interrupting male of status 1 and an interrupted male of status 2). These differences may be important, for example when dominance has nonlinear effects. To consider this possibility, we also conducted additional models, analyzing variation in “proportion of mating attempts interrupted,” “number of aggressive events,” and “aggression level of interruption,” in which we entered status as six levels: 1 versus 2 (1[2]), 1 versus 3 (1[3]), 2 versus 1 (2[1]), 2 versus 3 (2[3]), 3 versus 1 (3[1]), and 3 versus 2 (3[2]) in separate models. We found that this alternative approach produced very similar results (Table S2).

Our experimental design was an intermediate between nested and fully crossed, where a female subject is partially crossed with a male individual (not all combinations were used and during a trial, not all combination of individuals interacted; e.g. male A interrupted the mating attempt of male B on female C; male A never attempted to mate with female C). Because the degree of crossing was limited, we adopted a nested approach. In the analyses of “proportion of mating attempts interrupted” and “aggression level of interruption,” “female identity” nested within “attempting male identity” nested within “interrupting male identity” nested within “trial” was entered as a random factor. Similarly, we analyzed “number of aggressive interactions” with a GLMM in which “aggressor identity,” nested within “recipient identity,” nested within “trial” was entered as a random effect. The “aggressor” was defined as the individual that chased or won in a fight with the “recipient.” Using an alternative analysis based on a crossed approach produces qualitatively similar results, except for the “number of aggressive events,” where relatedness had an effect when random effects are assumed to be crossed: more aggressive interactions occurred between unrelated males than related males (Table S2). The significance of the fixed factors was assessed using the likelihood‐ratio test on models with and without the fixed factor (Valdar et al. 2006; Öckinger et al. 2010). We also tested the effect of “male type” (related or unrelated) on the “courtship counts before attempt” and “number of male‐initiated attempts” using two separate GLMMs with Poisson error distribution. “Male type” (R or U) and “dominance” (1 – 3; 1 being the most dominant) were entered as independent variables and “male type/dominance” was entered as an interaction. “Year” (1 or 2) was entered as a fixed factor. “Female identity” nested within “attempting male identity” nested within “trial” was entered as a random factor.

#### Female preference

We analyzed variation in female response through GLMMs with “male identity” nested within “female identity” nested within “trial” as a random factor, “year” (1 or 2) as a fixed factor, “male type” (R or U) and “dominance” (1 – 3; 1 being the most dominant) as fixed factors and “male type/dominance” as an interaction. The two response variables entered in two separate GLMMs were “proportion of attempts resisted,” and “probability of solicitation” both using a Binomial error distribution. “Average female resistance” was analyzed with a cumulative link‐mixed model. We also tested variation in the proportion of mating attempts performed by a male toward a female that was successful, through a GLMM with Binomial error distribution with mating success (yes or no) as the response variable.

All GLMM models were checked for over‐ or underdispersion. In Poisson‐distributed data, we added an observation‐level random factor in the model whenever overdispersion was detected (McCullagh and Nelder 1983; Harrison 2014). For cumulative link‐mixed models, we verified that the models fulfilled the proportional odds assumption.

#### Sperm allocation experiment

We tested whether males respond differentially to related and unrelated competitor males through two separate GLMMs with “male relatedness” (relatedness of focal male to first male), “relative dominance” (dominance of focal male relative to dominance of nonfocal male) and “female age” as fixed factors, “relatedness/relative dominance” as an interaction, “treatment order” (whether order A or B was 1st or 2nd) as a covariate and “focal male identity” and “trial” as crossed random factors. The response variables used in two separate GLMMs were “probability of spermic copulation” (yes or no) using a Binomial error distribution, and “number of sperm invested” using a Normal error distribution. The latter attained a Normal error distribution after a power ¼ transformation. To verify that the differences in sperm numbers were not due to aspermic copulations (successful copulations without sperm), we conducted an additional GLMM on the “number of sperm invested” in which we removed males that did not produce sperm in one of the trials. The significance of the fixed factor “relatedness” was assessed using the likelihood‐ratio test on models with and without the fixed factor.

#### Cryptic female choice experiment

We tested whether—after controlling for male factors (e.g., number of sperm inseminated)—we could detect patterns of sperm performance that could be parsimoniously explained by differential sperm utilization by the female after mating. We used three analyses to test the idea that females differentially select the sperm of the focal male depending on whether he was related or unrelated to the first male. First, we analyzed variation in the probability that sperm ejection was observed (“risk of sperm ejection”, Dean et al. 2011) with a GLMM with Binomial error distribution, “male relatedness,” “relative dominance,” and “female age” as fixed factors, “relatedness/relative dominance” as an interaction, and focal male identity’ nested within “female identity” as a random factor. Nesting took into account the fact that focal males were exposed to a different set of males and a different female in the paired design (Fig. 1C). Because the risk of sperm ejection is higher with larger ejaculates (Dean et al. 2011), we entered as a covariate the average volume of ejaculate produced by the focal male, which was measured by averaging the ejaculate volumes invested by the male in trials where he was used as the first male (2–4 estimates per male, mean ± SE: 2.4 ± 0.2). These trials were conducted at least 48 h apart, allowing for the complete replenishment of extragonadal sperm reserves (Etches 1996), thus avoiding the risk that sperm depletion caused by successive copulations would result in temporal declines in the volume of ejaculates produced by a male. We measured the proportion of an ejaculate that was ejected by a female (“intensity of sperm ejection”, Dean et al. 2011), using this average ejaculate volume as an estimate of the volume delivered. We analyzed variation in the intensity of sperm ejection with a Mann–Whitney *U* test with “male relatedness” and “relative dominance” as fixed factors. No interaction between relatedness and dominance could be tested for this response because of the limited sample size. Finally, we analyzed patterns of female sperm utilization by quantifying variation in PVL hydrolysis points using a GLMM with Poisson error distribution, “male relatedness,” “relative dominance,” and “female age” as fixed factors, “relatedness/relative dominance” as interaction terms, and “focal male identity” nested within “female identity” as a random factor. Because all else being equal, the number of sperm reaching individual eggs is a function of the number of sperm initially inseminated, we included the average number of sperm that the focal male delivered as first male as a covariate.

## Results

### PRECOPULATORY EXPERIMENT

#### Male–male competition

**Figure 2:**
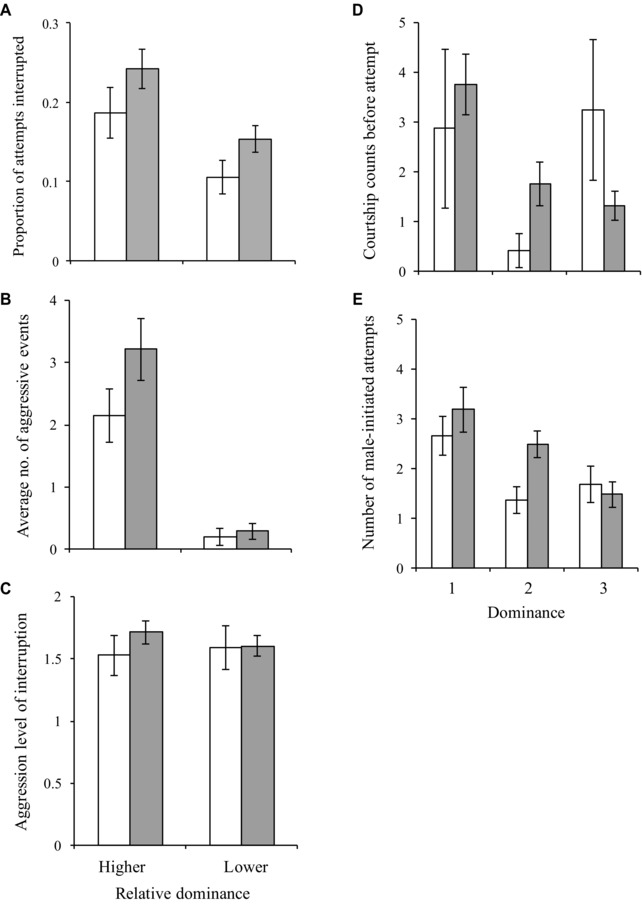
Precopulatory male response to related (white bars) and unrelated (gray bars) rivals. Error bars denote SE. (A) Males interrupted on average a significantly higher proportion of mating attempts by unrelated rivals than by related rivals. (B) There was a non‐significant tendency for a higher number of aggressive events between unrelated males than between related males. (C) There was no significant difference in the level of aggression displayed in interruptions between unrelated males and between related males. On the *x*‐axis of (A)‐(C) is the dominance status of the interrupter relative to the dominance status of the attempting male. (D) There was no significant difference in the frequency at which related and unrelated males courted individual females. (E) There was no significant difference in the number of mating attempted on average by related and unrelated males with individual females. On the *x*‐axis of (D) and (E) is male social status.

We first tested whether male status was influenced by relatedness among males. We found no evidence of this, the single unrelated male was equally likely to occupy top‐, intermediate, and bottom rank (Table S3; all untransformed estimates of random factors and covariates as well as the mean and standard errors of each combination of relatedness and dominance levels are presented in Table S4, S5, and S6, respectively). After statistically accounting for the effect of status, a significantly higher per male proportion of mating attempts was interrupted by a male unrelated (rather than related) to the mating male (Table 1(i)a; Fig. 2A). Interruptions between unrelated males can be categorized as two types: when a related male interrupts the unrelated male (RU) and when the unrelated male interrupts the related male (UR). The third type of interaction is interruptions between related males (RR). To establish whether mating interruptions among unrelated males were caused by RU or UR, we conducted a second analysis of “proportion of mating attempts interrupted” with “relatedness” as a fixed factor with three levels (RU, UR, RR). This confirmed that RU > UR = RR (Relatedness, χ^2^_1_ = 7.066, *P* = 0.029; Post‐hoc: RU – RR, *Z* = 2.378, *P* = 0.044; UR – RR, *Z* = 1.954, *P* = 0.121; UR – RU, *Z* = 0.292, *P* = 0.953), indicating that the two related males directed their interruptions preferentially toward the unrelated male rather than toward each other. There was also a nonsignificant tendency for a higher number of aggressive events to occur between unrelated males than between related males (Table 1(i)b; Fig. 2B). To establish the extent to which this was caused by related males aggressing the unrelated male (RU) or vice versa (UR), we conducted an analysis of aggressive events among RU, UR, and RR categories (as outlined above), which suggested that UR>RU = RR (Relatedness, χ^2^_1_ = 6.325, *P* = 0.042; Post‐hoc: RU – RR, *Z* = –0.101, *P* = 0.994; UR – RR, *Z* = 2.313, *P* = 0.053; UR – RU, *Z* = 1.980, *P* = 0.116). There was no difference in the aggression level of interruption of related and unrelated males (Table 1(i)c; Fig. 2C).

**Table 1:** Results of the precopulatory experiment

Response variable	Factors	Values (mean ± SE)	df	Test statistic	*P*
(i) Male behavior					
(a) Proportion of mating attempts interrupted	Relatedness	R: 0.139 ± 0.018; U: 0.187 ± 0.014	1	6.980	**0.008**
	Relative dominance	H: 0.223 ± 0.020; L: 0.139 ± 0.013	1	21.153	**<0.001**
	Relatedness: Relative dominance		1	3.065	0.690
(b) Number of aggressive events	Relatedness	R: 1.187 ± 0.249; U: 1.743 ± 0.278	1	2.812	0.094
	Relative dominance	H: 2.851 ± 0.361; L: 0.259 ± 0.099	1	67.611	**<0.001**
	Relatedness: Relative dominance		1	0.002	0.965
(c) Aggression level of interruption	Relatedness	R: 1.556 ± 0.116; U: 1.657 ± 0.062	1	0.247	0.805
	Relative dominance	H: 1.662 ± 0.081; L: 1.600 ± 0.075	1	0.277	0.782
	Relatedness: Relative dominance		1	0.040	0.968
(d) Courtship counts before attempt	Relatedness	R: 2.341 ± 0.780; U: 2.280 ± 0.288	1	3.099	0.078
	Dominance	1: 3.479 ± 0.643; 2: 1.417 ± 0.346;	2	7.713	**0.021**
		3: 2.000 ± 0.543			
	Relatedness: Dominance		2	3.002	0.223
(e) Number of male‐initiated attempts	Relatedness	R: 1.950 ± 0.218; U: 2.431 ± 0.198	1	0.253	0.615
	Dominance	1: 2.964 ± 0.307; 2: 2.176 ± 0.209;	2	11.466	**0.003**
		3: 1.583 ± 0.221			
	Relatedness: Dominance		2	2.292	0.318
(ii) Female response					
(a) Proportion of mating attempts resisted	Relatedness	R: 0.616 ± 0.026; U: 0.535 ± 0.035	1	7.104	**0.008**
	Dominance	1: 0.608 ± 0.031; 2: 0.559 ± 0.037;	2	1.119	0.572
		3: 0.585 ± 0.043			
	Relatedness: Dominance		2	0.764	0.683
(b) Average female resistance	Relatedness	R: 3.625 ± 0.070; U: 3.202 ± 0.131	1	14.78	**<0.001**
	Dominance	1: 3.320 ± 0.072; 2: 3.201 ± 0.083;	2	2.205	0.332
		3: 3.300 ± 0.104			
	Relatedness: Dominance		2	1.557	0.459
(c) Probability of solicitation	Relatedness	R: 0.012 ± 0.008; U: 0.119 ± 0.036	1	12.356	**<0.001**
	Dominance	1: 0.024 ± 0.017; 2: 0.071 ± 0.028;	2	2.018	0.365
		3: 0.048 ± 0.023			
	Relatedness: Dominance		2	3.790	0.150
(iii) Proportion of mating attempts that were successful					
	Relatedness	R: 0.279 ± 0.045; U: 0.498 ± 0.076	1	4.775	**0.029**
	Dominance	1: 0.440 ± 0.075; 2: 0.343 ± 0.063;	2	1.860	0.395
		3: 0.227 ± 0.057			
	Relatedness: Dominance		2	1.592	0.451

R = related, U = unrelated male; H = higher, L = lower social status of the focal male (interrupter or aggressor) compared to the recipient male; 1 = top‐, 3 = bottom‐ranking male. Test statistic values are based on the χ^2^‐distribution, except for aggression level of interruption, which is based on the *Z*‐distribution. *P* < 0.05 are highlighted in bold.

#### Female preference

**Figure 3:**
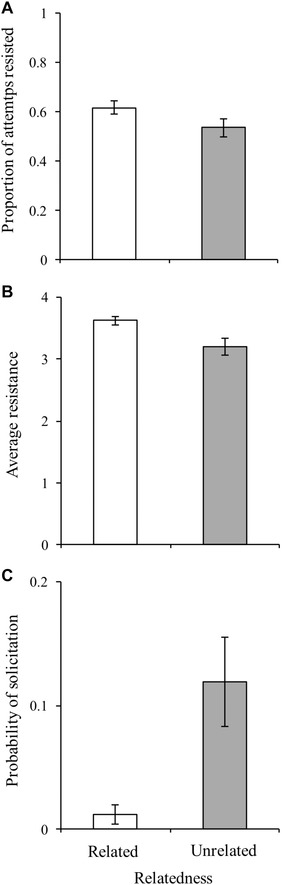
Female response to related (R) (white bars) and unrelated (U) (gray bars) male types. Error bars denote SE. (A) There was a significantly higher female resistance to mating attempts by R males than by the U male. (B) Females displayed a significantly higher probability of soliciting to the U male than either of the R males. (C) Females resisted a significantly higher proportion of mating attempts by R males than by the U male.

**Figure 4:**
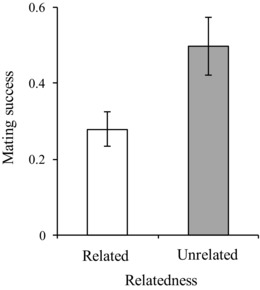
Proportion of male‐initiated mating attempts with individual females that were successful across related and unrelated male types. A significantly higher proportion of mating attempts by the unrelated male led to a successful copulation compared to each of the two related males.

After statistically accounting for dominance, females resisted proportionally less (Table 1(ii)a; Fig. 3A), and displayed a lower average level of resistance toward the mating attempts of the unrelated male in the group (Table 1(ii) b; Fig. 3B). Females were also more likely to solicit copulation from the unrelated male than from either of the two related males (Table 1(ii)c; Fig. 3C), despite the fact that there was no significant difference in the number of courtship or male‐initiated attempts performed by the related males or by the unrelated male (Table 1(i)d, 1(i)e; Fig. 2D, E). Largely as a result of this female preference, a significantly higher proportion of mating attempts by the unrelated male was successful as compared to either related male (Table 1(iii)a; Fig. 4).

### POSTCOPULATORY EXPERIMENTS

#### Male sperm allocation in sperm competition

**Figure 5:**
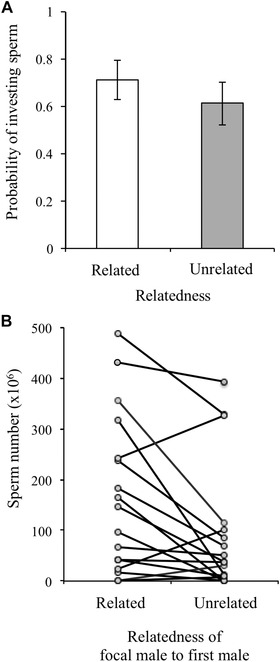
Male sperm allocation in response to sperm competition with related and unrelated rivals. Focal males were allowed to copulate with a female fitted with a harness after witnessing a related rival or an unrelated rival copulating with the female. (A) There was no significant difference in the probability of investing sperm between related and unrelated male types. (B) Focal males invested significantly more sperm in the female that was first mated to the related rather than the unrelated male. Each line represents the response of individual males, some of which are averaged over multiple trials.

There was no significant difference in the probability that a focal male performed a spermic copulation with a female after she had mated with a first related or unrelated male (Table 2(i)a; Fig. 5A). Relatedness, however, had a significant effect on the number of sperm that a male invested in a female. Contrary to expectations, males invested significantly more sperm in a female after she had mated with a related male rather than after she had mated with the unrelated male (Table 2(i)b; Fig. 5B).

**Table 2:** Results of the postcopulatory experiments

Response variable	Factors	Values (mean ± SE)	df	Test statistic	*P*
(i) Male response					
(a) Probability of investing sperm	Relatedness	R: 0.710 ± 0.083; U: 0.613 ± 0.089	1	0.993	0.319
	Relative dominance	H: 0.630 ± 0.095; L: 0.686 ± 0.080	1	0.225	0.635
	Relatedness: Relative dominance		1	0.263	0.608
(b) Amount of sperm invested	Relatedness	R: 2.14 ± 0.44; U: 1.29 ± 0.38	1	5.868	**0.015**
in 10^8^ of sperm	Relative dominance	H: 1.26 ± 0.29; L: 2.08 ± 0.46	1	0.587	0.444
(with aspermic attempts)	Relatedness: Relative dominance		1	0.095	0.758
(c) Amount of sperm invested	Relatedness	R: 2.32 ± 0.58; U: 1.43 ± 0.44	1	4.592	**0.032**
in 10^8^ of sperm	Relative dominance	H: 1.11 ± 0.33; L: 2.33 ± 0.53	1	0.767	0.381
(without aspermic attempts)	Relatedness: Relative dominance		1	1.567	0.211
(ii) Female response					
(a) Probability of sperm ejection	Relatedness	R: 0.235 ± 0.106; U: 0.150 ± 0.082	1	0.604	0.437
	Relative dominance	H: 0.167 ± 0.078; L: 0.231 ± 0.122	1	0.391	0.532
	Relatedness: Relative dominance		1	0.285	0.593
(b) Proportion of semen ejected	Relatedness	R: 0.573 ± 0.128; U: 0.171 ± 0.030	1	12	0.057
	Relative dominance	H: 0.428 ± 0.124; L: 0.363 ± 0.219	1	8	0.629
(c) Number of hydrolysis points	Relatedness	R: 4.77 ± 1.10; U: 7.43 ± 2.35	1	0.649	0.426
	Relative dominance	H: 8.20 ± 2.45; L: 3.81 ± 0.59	1	0.454	0.500
	Relatedness: Relative dominance		1	0.454	0.500
	Relatedness: Lay date		1	3.605	0.058

R = related, U = unrelated male; H = higher, L = lower social status of the focal male compared to the first male to mate with the same female. Test statistic values are based on the χ^2^‐distribution, except for aggression level of interruption, which is based on the Z‐distribution and the proportion of semen ejected that is based on the Mann–Whitney U test statistic. *P* < 0.05 are highlighted in bold.

#### Cryptic female choice

**Figure 6:**
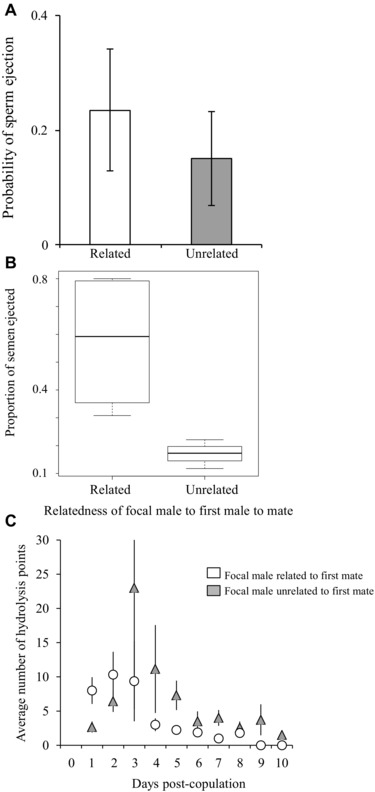
Postcopulatory female response to related and unrelated male types. Females fitted with harnesses were first mounted by a nonfocal male, then allowed to copulate without the harness with a focal male that was either related or unrelated to the first male. Error bars denote SE. (A) There was no difference in the probability of sperm ejection suffered by focal males related to first males and by focal males unrelated to first males. (B) Females ejected a marginally nonsignificantly higher proportion of semen from focal males related to first males than from focal males unrelated to first males. (C) Eggs laid on the first day contained more sperm‐induced hydrolysis points after copulation with a focal male related to the first male to mate than with a focal male unrelated to the first male. However, from the third day of the laying sequence onwards eggs produced following mating with unrelated focal males tended to contain more hydrolysis points.

The risk of sperm ejection was generally low and there was no difference in the number of females ejecting sperm from related or unrelated males (four from related, and three from unrelated males; Table 2(ii)a; Fig. 6A). In the seven trials in which semen ejection did occur, there was a marginally nonsignificant tendency for females to eject a lower proportion of sperm when the focal male was unrelated (Table 2(ii)b; Fig. 6B).

We then considered patterns of sperm‐induced hydrolysis points on the egg PVL. The null hypothesis (females do not bias sperm utilization) predicts that more PVL hydrolysis points are found on the eggs when the focal male is related to the first male, because our sperm allocation results indicate that males inseminate relatively more sperm in this role (Table 2(i)b; Fig. 5B), which means that more sperm should reach the eggs. The alternative hypothesis is that females do control sperm utilization and bias it in response to within‐group male relatedness. In principle, this bias could favor the focal male either when he is related or when unrelated to the first male. The prediction of the former scenario is aligned with (i.e., difficult to tease apart from) the prediction of the null hypothesis. The latter scenario, however, predicts fewer sperm‐induced hydrolysis points on the eggs when the focal male is related to the first male than one would expect based solely on the fact that males inseminate more sperm in this role. We found that the influence of male relatedness on the number of sperm‐induced hydrolysis points of the egg changed over the laying cycle. While on the first two days of lay there were more hydrolysis points in the eggs following copulation with the related male (broadly consistent with the null hypothesis), this pattern was reversed over subsequent days of the laying cycle, when eggs produced following mating with the unrelated male tended to contain more hydrolysis points, resulting in a marginally nonsignificant day/relatedness interaction effect (Table 2(ii)b; Fig. 6C). Collectively, these results show that variation in the number of sperm reaching individual eggs is not entirely explained by number of sperm inseminated alone, and we cannot rule out a possible–albeit weak—female bias in favor of the inseminations of unrelated males from the third day of lay onwards.

## Discussion

Relatedness among competing males has long been recognized as a potential key factor in the operation of sexual selection in structured populations. Tests of this idea, however, have largely focused on precopulatory male–male competition, ignoring other mechanisms of sexual selection. This study set out to conduct an experimental investigation of the role of within‐group male relatedness across both pre‐ and postcopulatory mechanisms of sexual selection in small social units of a polyandrous population of red junglefowl. We show that in this population, the degree of relatedness between rival males modulates the intensity of male competition and influences female responses. The intensity of precopulatory competition was reduced between related males, with fewer occurrences of mating interruptions and aggressive events. Females on the other hand, biased mating in favor of the single male who was unrelated to the two closely related males in the group. This female bias appears to have considerable impact on a male's ability to mate successfully with a female, given that a higher proportion of mating attempts by the unrelated male was successful, independently of his social status and despite reduced precopulatory competition among related males. Within‐group male relatedness also played a role in postcopulatory sexual selection: while patterns of cryptic female choice were weak and ambiguous, males invested more sperm in sperm competition with a relative, contrary to theoretical expectations.

The observation of reduced precopulatory competition among related males by this study is consistent with preferential cooperation among related males reported in a number of species (Pizzari et al. 2015; Kapranas et al. 2016), including some populations of *Drosophila melanogaster* (Carazo et al. 2014, 2015; Martin and Long 2015; but see Chippindale et al. 2015), and other galliformes, such as peacocks, *Pavo cristatus* (Petrie et al. 1999), and wild turkeys, *Meleagris gallopavo* (Krakauer 2005). These patterns are broadly in line with inclusive fitness theory, which predicts that social behaviors reducing the personal fitness of an actor can evolve when direct costs are compensated by indirect fitness benefits when actor and recipient are sufficiently more related to each other than the population average (Hamilton 1964). If a male is unable to monopolize all females completely, which is almost invariably the case in multimale, multifemale groups of fowl (e.g., Collet et al. 2012), it is conceivable that a male would benefit by sharing females with a related rather than an unrelated male. This would be especially relevant when males related to each other are disadvantaged in competition with unrelated males, for example because of female preference (see below).

Our study also indicates that females respond differentially to within‐group male relatedness in a way that may counteract cooperation between male relatives. One possible explanation may be that female preference for unrelated males reflects a more general preference for rare male genotypes (O'Donald 1977; Partridge 1988). Evidence consistent with the rare male effect has been found by a number of studies (Singh and Sisodia 2000; Hughes et al. 2013), although alternative interpretations have proven difficult to rule out. For example, the rare male may compete more vigorously for females or become more sexually active to compensate for cooperation among related males (Knoppien 1985; Partridge 1988). Here, we differentiated the relative contribution of these factors and show that male activity played a minimal role in the rare male advantage: the related and unrelated males did not differ in the number of attempts or courtship events, suggesting that in this case female red junglefowl may actively bias mating in favor of rare male types. The adaptive significance of this preference remains unclear, but may increase the genetic diversity of a brood, by favoring paternity of sires that are less genetically similar to each other (Fossøy et al. 2007; Jennions and Petrie 2007). Preference for males with a rare genotype might also aid in inbreeding avoidance. In our experiments, females were always unrelated to males. However, in populations where dispersal is limited in both sexes, the existence of related males within a group may also increase the chances of females being related to the males, and avoidance of related males could be a mean of avoiding the costs of inbreeding. Alternatively, female behavior might be the consequence of avoidance of previous mates. Females have been shown to prefer sexually novel males in some promiscuous mating systems (Lisk and Baron 1982; Bateman 2004; but see Tan et al. 2013). A preference for genetically different mates would then enable females to reduce the risk of mating repeatedly with the same male.

Our study also explored whether females might bias sperm utilization in response to male–male relatedness. Patterns of sperm ejection are consistent with those observed in a previous study for females exposed to two successive matings, where the risk of sperm ejection is relatively low (Dean et al. 2011). There was a nonsignificant trend for females to eject a lower proportion of ejaculates from unrelated males in the few cases when sperm ejection was confirmed. Patterns of sperm‐induced hydrolysis points on the eggs suggested that—after the first two days postinsemination—ejaculates of unrelated males were marginally more represented on the PVL. Overall, however, these patterns are statistically weak, especially after controlling for multiple testing (sperm ejection risk, sperm ejection intensity, sperm‐induced hydrolysis points). Sample sizes are limited and some of the models have many explanatory variables (models of sperm ejection risk and sperm‐induced hydrolysis points), which limits the power to detect small effect sizes. In addition, it is difficult to extrapolate how such possible female bias emerging only two days after the insemination may affect male reproductive success in the complexity of natural populations where females are likely to have received new inseminations (from the same male or from other males) in the intervening time. It is possible that, all else being equal, a female tendency to bias sperm utilization against the sperm of related males might mean that a male may obtain a lower share of paternity for a standard unit of sperm investment when mating after his relative. A study of *Drosophila melanogaster* in which two males related to each other competed with an unrelated male over access to females, reported that the unrelated male had a disproportionate share of paternity (Carazo et al. 2014). Given that flies interacted and mated freely, these patterns may reflect female‐driven mechanisms (i.e., cryptic female choice for rare males, Pizzari et al. 2015), male‐driven mechanisms, or a combination of the two. The results of our present study demonstrate the need to consider pre‐ and postcopulatory female responses when studying the role of within‐group male relatedness in sexual selection and sexual conflict. In fact, the contrasting role of male relatedness in precopulatory male–male competition and female preference may shed new light on sexual conflict. Theory predicts that in structured populations, high local male relatedness can reduce male harm of females thus reducing conflict (Wild et al. 2011; Faria et al. 2015). However, if females benefit by mating with males genetically different from each other, while males benefit by sharing females with their own relatives, there will be sexual conflict over the relatedness of a female's partners. Within‐group male relatedness may in this case increase, rather than relax, sexual conflict over mating and fertilization, suggesting that the role of within‐group male relatedness in sexual conflict can be complex and is likely to change dynamically with patterns of female preference and across time.

Our study indicates that male red junglefowl respond to the relatedness of their sperm competitors, but in a way that is contrary to predictions of ejaculate economic theory (Parker 2000): males allocated more rather than fewer sperm when competitors were related. The few empirical studies to investigate male sperm allocation strategies in response to their relatedness with competitor males have largely failed to demonstrate a differential response (Thomas and Simmons 2008; Ramm and Stockley 2009). The adaptive significance of these male responses is unclear. Resolving this challenge will require consideration of a number of factors. First, there may be differential costs of male pre‐ versus postcopulatory competition and within‐group male relatedness may modulate the trade‐off between male expenditures in pre‐ and postcopulatory competition (Parker et al. 2013). In this species, male fights in precopulatory competition are well known to result in long‐term costs, such as loss of an eye or leg injuries and even death (Craig 1981; T. Pizzari, pers. obs.), while sperm competition is not associated with risk of injuries. If male investment in postcopulatory competition is traded off against investment in precopulatory competition (Parker et al. 2013), it is possible that when males compete preferentially with relatives, they may invest less in precopulatory competition. This may leave them with more resources to allocate to postcopulatory competition. A second contributing factor may be the differential relatedness of pre‐ versus postcopulatory competitors. In other words, a male's relatedness to a competitor relative to the average relatedness in the group will change between pre‐ and postcopulatory competition if within‐group male relatedness influences the subset of males that will successfully mate with a female and compete after copulation. These changes will modulate inclusive fitness consequences of male investment in pre‐ and postcopulatory competition. Finally, it is possible that patterns of possible cryptic female choice in relation to within‐group male relatedness may also affect male strategies of sperm allocation (Ball and Parker 2003).

As in all studies of strategic sperm allocation, an important caveat is that male responses are likely to be somewhat contingent on the socio‐sexual cues simulated by the experiment. In this study, we adopted an established experimental design that seeks to control for variation in sperm numbers due to male sperm depletion, by limiting the frequency at which experimental males were exposed to mating opportunities. It is therefore possible that the experimental removal of sperm depletion may have influenced male patterns of sperm allocation. This seems however unlikely. First, males had little opportunity to learn the experimental mating frequency and predict when they would mate next. In this species the first copulation with a female tends to deliver a very large proportion of a male's extragonadal sperm reserves (Pizzari et al. 2003), which take ∼48 hours of sexual rest to replenish completely (Etches 1996). This means that even a single mating can impact on a male's chances of fertilization over the next 1–2 days, particularly when sperm competition is as intense as in these groups. Second, it is not clear how the pattern of female availability imposed by the experiment would have resulted in a preferential investment in competition with related males. In the future, it will be important to explore the way these responses change across different regimes of male competition and female availability.

Finally, our results also confirm previous work indicating kin recognition in red junglefowl (Pizzari et al. 2004; Løvlie et al. 2013). Specifically, we reveal evidence for two patterns of kin recognition: female recognition of male relatedness and male recognition of his own relatedness to other males. The former may be achieved if females were able to identify the single unrelated male through sensory habituation to the cue of the common type of male (Ehrman and Spiess 1969). The “sensory habituation” hypothesis proposes that females habituate to the stimulus of the common male type and respond more strongly to the different stimulus of the rare type (Ehrman and Spiess 1969). The proximate explanation of male behavior, however, requires classic kin recognition. Two widely discussed mechanisms are: prior association, where kin discrimination is based on social familiarity, and phenotype matching, where recognition is based on self‐referent cues (Holmes and Sherman 1982; Holmes 1986). The fact that the study birds were artificially hatched and raised in batches containing siblings and unrelated individuals suggest that social familiarity alone cannot explain kin recognition in this species. This is consistent with previous work, indicating that kin recognition may be due to innate mechanisms, such as self‐reference phenotype‐matching, in other species of galliformes (Bateson 1982; Waldman and Bateson 1989; Petrie et al. 1999). It is, however, possible that the mechanism at work might be more complex. For example, kin recognition may require an interaction between social familiarity and genetic relatedness per se. Future work should seek to resolve the specific proximate mechanisms underpinning kin recognition in this species, and their ontogenetic development.

In conclusion, we show that within‐group male relatedness can have considerable but contrasting effects in multiple mechanisms of sexual selection. These effects can counteract each other and are not always easily explained by current theory. Our results therefore provide a proof of the concept that studies of sexual selection, particularly those investigating structured populations, should consider multiple roles that relatedness may play, and similarly that sexual selection theory should be expanded to resolve the complexity of these effects. Further research is needed to determine the fitness consequences of these behaviors and to uncover the underlying mate recognition mechanisms.

Associate Editor: R. Rodriguez

Handling Editor: M. Servedio

## Supporting information


**Table S1**. Expected and observed heterozygosities (H_e_ and H_o_), allele sizes and allelic frequencies of the 26 microsatellite loci used to genotype the red jungle fowl.
**Table S2**. Results of the separate analyses considering random factors as crossed and dominance as 6 levels: 1 versus 2 (1[2]), 1 versus 3 (1[3]), 2 versus 1 (2[1]), 2 versus 3 (2[3]), 3 versus 1 (3[1]), 3 versus 2 (3[2]).
**Table S3**. Number of trials in which the single unrelated male exhibited a particular status.
**Table S4**. Untransformed estimates of fixed effects and covariates.
**Table S5**. Effects of random factors and covariates. (A) Precopulatory experiments. (B) Postcopulatory experiments. E‐08 indicate 10^−8^ etc. Female response proportion of semen ejected is not shown as there were only 7 samples where sperm was ejected and we used a Mann‐Whitney U test.
**Table S6**. Means and SE of each level of relatedness and dominance combination. (A) Precopulatory experiments. (B) Postcopulatory experiments. Columns are separated by dominance status. Female response proportion of semen ejected is not shown as there were only 7 samples where sperm was ejected.

**Figure S1**. Male hierarchy on day 0 and during experiment.

**Figure S2**. Repeatability of male aggression behaviour across trials.
