# Supplementary material for: The contrasting role of male relatedness in different mechanisms of sexual selection in red junglefowl
Source: Evolution. 2017 Jan 5;71(2):403–20. doi: 10.1111/evo.13145 (PMC5324671; doi:10.1111/evo.13145)
Supplement: Supplementary file 1 — Table S1. Expected and observed heterozygosities (He and Ho), allele sizes and allelic frequencies of the 26 microsatellite loci used to genotype the red jungle fowl. Table S2. Results of the separate analyses considering random factors as crossed and dominance as 6 levels: 1 versus 2 (1[2]), 1 versus 3 (1[3]), 2 versus 1 (2[1]), 2 versus 3 (2[3]), 3 versus 1 (3[1]), 3 versus 2 (3[2]). Table S3. Number of trials in which the single unrelated male exhibited a particular status. Table S4. Untransformed estimates of fixed effects and covariates. Table S5. Effects of random factors and covariates. (A) Precopulatory experiments. (B) Postcopulatory experiments. E‐08 indicate 10−8 etc. Female response proportion of semen ejected is not shown as there were only 7 samples where sperm was ejected and we used a Mann‐Whitney U test. Table S6. Means and SE of each level of relatedness and dominance combination. (A) Precopulatory experiments. (B) Postcopulatory experiments. Columns are separated by dominance status. Female response proportion of semen ejected is not shown as there were only 7 samples where sperm was ejected. [file EVO-71-403-s001.docx]

Table S1. Expected and observed heterozygosities (H_e_ and H_o_), allele sizes and allelic frequencies of the 26 microsatellite loci used to genotype the red jungle fowl. All Individuals were genotyped at between 16 and 26 loci (Median = 16).

| **Locus** | **Exp Hz** | **Obs Hz** | **Allele** | **Freq** | **Reference** |
| --- | --- | --- | --- | --- | --- |
| **ADL0180** | 0.779 | 0.694 | 130  132  138  142  144 | 0.062  0.250  0.243  0.257  0.187 | Cheng et al. 1994 |
| **ADL0183** | 0.597 | 0.616 | 95  99  105  107  109  119 | 0.603  0.103  0.061  0.157  0.068  0.007 | Cheng et al. 1994 |
| **ADL0188** | 0.67 | 0.603 | 140  151  152  154 | 0.192  0.055  0.452  0.301 | Cheng et al. 1994 |
| **ADL0299** | 0.686 | 0.500 | 130  132  150  158  162 | 0.333  0.167  0.417  0.076  0.007 | Cheng et al. 1994 |
| **LEI0028** | 0.732 | 0.671 | 153  159  161  167  173 | 0.384  0.288  0.116  0.164  0.048 | Hanotte et al. 1997 |
| **LEI0068** | 0.688 | 0.616 | 221  227  229  231  235 | 0.342  0.404  0.178  0.018  0.062 | Gibbs et al. 1995 |
| **LEI0078** | 0.66 | 0.425 | 328  338  436  461  463  487 | 0.007  0.329  0.123  0.069  0.466  0.007 | Gibbs et al. 1997 |
| **LEI0095** | 0.551 | 0.370 | 319  325  329  349 | 0.431  0.514  0.009  0.048 | Gibbs et al. 1997 |
| **LEI0109** | 0.619 | 0.534 | 204  206  208 | 0.151  0.452  0.397 | Gibbs et al. 1997 |
| **LEI0127** | 0.719 | 0.575 | 222  224  232  248  254  256 | 0.034  0.384  0.075  0.343  0.062  0.103 | Gibbs et al. 1997 |
| **LEI0196** | 0.742 | 0.575 | 170  172  174  180  182  184  186  190  192 | 0.110  0.157  0.014  0.027  0.034  0.459  0.048  0.089  0.062 | Dawson et al. 1998 |
| **LEI0246** | 0.825 | 0.822 | 207  225  229  241  245  249  261  265  269  273  455 | 0.157  0.027  0.014  0.096  0.014  0.336  0.068  0.137  0.014  0.068  0.068 | McConnell et al. 1999 |
| **MCW0014** | 0.696 | 0.278 | 172  178  182  186 | 0.292  0.056  0.264  0.389 | Crooijmans et al. 1996 |
| **MCW0123** | 0.666 | 0.493 | 77  79  81  85  87 | 0.075  0.301  0.137  0.473  0.014 | Crooijmans et al. 1996 |
| **MCW0183** | 0.693 | 0.556 | 292  300  304  316 | 0.306  0.333  0.326  0.035 | Crooijmans et al. 1997 |
| **ROS0081*** | 0.739 | 0.548 | 307  309  311  313  315  317 | 0.390  0.007  0.116  0.192  0.247  0.048 | Groenen et al. 2000 |
| **ADL0268** | 0.393 | 0.306 | 105  107  111 | 0.098  0.764  0.139 | Cheng et al. 1994 |
| **ADL0138** | 0.476 | 0.429 | 109  111  113  117  123  129 | 0.057  0.100  0.714  0.072  0.043  0.014 | Cheng et al. 1994 |
| **LEI0118** | 0.433 | 0.278 | 165  169  171  173 | 0.028  0.028  0.222  0.722 | Gibbs et al. 1997 |
| **LEI0192** | 0.547 | 0.429 | 244  257  267  271  331 | 0.027  0.186  0.643  0.043  0.100 | McConnell et al. 1999 |
| **LEI0251** | 0.641 | 0.563 | 101  105  113  119 | 0.531  0.281  0.094  0.094 | Dawson et al. 1998 |
| **LEI0194** | 0.669 | 0.357 | 125  126  133  154  158 | 0.536  0.071  0.107  0.214  0.071 | McConnell et al. 1999 |
| **LEI0223** | 0.699 | 0.800 | 181  185  209  213  215 | 0.1667  0.1667  0.0333  0.1333  0.5000 | Dawson et al. 1998 |
| **MCW0330** | 0.669 | 0.813 | 268  276  286 | 0.250  0.438  0.313 | Crooijmans et al. 1997 |
| **LEI0237** | 0.7 | 0.750 | 227  309  318  334  351 | 0.063  0.031  0.250  0.188  0.469 | McConnell et al. 1999 |
| **LEI0328** | 0.593 | 0.438 | 381  405  417  421 | 0.156  0.594  0.219  0.031 | McConnell et al. 1999 |

Gibbs M., D. A. Dawson, C. McCamley, and T. Burke. 1995 Ten novel chicken dinucleotide repeat polymorphisms. Animal Genetics 26:443–4.

Cheng, H.H., and L.B. Crittenden. 1994. Microsatellite markers for genetic mapping in the chicken. Poultry Science 73:539-546.

Gibbs, M., D. A. Dawson, C. McCamley, A. F. Wardle, J. A. L. Armour, and T. Burke. 1997. Chicken microsatellite markers isolated from libraries enriched for simple tandem repeats. Animal Genetics 28:401-417.

Dawson, D., S. McConnell, A. Wardle, M. Gibbs, and T. Burke. 1998. Characterization and mapping of 15 novel chicken microsatellite loci. Animal Genetics 29:159-160.

Crooijmans, R., R. Dijkhof, J. J. Poel, and M. Groenen. 1997. New microsatellite markers in chicken optimized for automated fluorescent genotyping. Animal Genetics 28:427-437.

Crooijmans, R. P. M. A., P. A. M. van Oers, J. A. Strijk, J. J. van der Poel, and M. A. M. Groenen. 1996. Preliminary linkage map of the chicken (Gallus domesticus) genome based on microsatellite markers: 77 new markers mapped. Poultry Science 75:746-754.

Hanotte, O., A. Pugh, C. Maucher, D. Dawson, and T. Burke. 1997. Nine novel chicken microsatellite loci and their utility in other Galliformes. Animal Genetics 28.

Groenen, M. A. M., H. H. Cheng, N. Bumstead, B. F. Benkel, W. E. Briles, T. Burke, D. W. Burt, L. B. Crittenden, J. Dodgson, and J. Hillel. 2000. A consensus linkage map of the chicken genome. Genome Research 10:137-147.

McConnell, S. K. J., Dawson, D., Wardle, A. and Burke, T. 1999. The isolation and mapping of nineteen tetranucleotide microsatellite markers in the chicken. Animal Genetics. 30:183–189.

**Table S2**. Results of the separate analyses considering random factors as crossed and dominance as 6 levels: 1 versus 2 (1[2]), 1 versus 3 (1[3]), 2 versus 1 (2[1]), 2 versus 3 (2[3]), 3 versus 1 (3[1]), 3 versus 2 (3[2]).

| PRECOPULATORY RESPONSE |  |  |  |  |  |  |  |  |  |  |  |
| --- | --- | --- | --- | --- | --- | --- | --- | --- | --- | --- | --- |
|  |  | Dominance coded as Higher versus Lower | | | |  | Dominance coded as 1,2,3 | | | | |
|  |  | Nested | Crossed | | | Nested | | | Crossed | | |
|  |  |  | *df* | Chi-sq | *p* | *df* | Chi-sq | *p* | *df* | Chi-sq | *p* |
| (i) Male behavior |  |  |  |  |  |  |  |  |  |  |  |
| Proportion of mating attempts interrupted | Relatedness | Results in Table 1 | 1 | 6.941 | **0.008** | 1 | 5.274 | **0.022** | 1 | 5.157 | **0.023** |
|  | Dominance (6 levels) |  | 1 | 26.357 | **<0.001** | 2 | 4.424 | **0.035** | 5 | 6.709 | **0.010** |
|  | Relatedness:Dominance |  | 1 | 0.902 | 0.970 | 2 | 0.113 | 0.737 | 5 | 0.560 | 0.454 |
|  |  |  |  |  |  |  |  |  |  |  |  |
| Number of aggressive events | Relatedness |  | 1 | 4.597 | **0.032** | 1 | 2.649 | 0.104 | 1 | 3.255 | 0.071 |
|  | Dominance (6 levels) |  | 1 | 109.900 | **<0.001** | 2 | 80.705 | **<0.001** | 5 | 123.960 | **<0.001** |
|  | Relatedness:Dominance |  | 1 | 0.192 | 0.661 | 2 | 4.889 | 0.430 | 5 | 5.281 | 0.383 |
|  |  |  |  |  |  |  |  |  |  |  |  |
| Aggression level of interruption | Relatedness |  | 1 | 0.129 | 0.897 | 1 | 1.301 | 0.193 | 1 | 1.262 | 0.207 |
|  | Dominance (6 levels) |  | 1 | 0.174 | 0.862 | 2 | 0.621 | 0.535 | 5 | 0.568 | 0.570 |
|  | Relatedness:Dominance |  | 1 | 0.263 | 0.792 | 2 | 1.242 | 0.214 | 5 | 1.333 | 0.183 |
|  |  |  |  |  |  |  |  |  |  |  |  |
| Courtship counts before attempt | Relatedness |  |  |  |  | Results in Table 1 | | | 1 | 2.728 | 0.099 |
|  | Dominance (3 levels) |  |  |  |  |  |  |  | 2 | 7.712 | **0.021** |
|  | Relatedness:Dominance |  |  |  |  |  |  |  | 2 | 3.004 | 0.223 |
|  |  |  |  |  |  |  |  |  |  |  |  |
| Number of male-initiated attempts | Relatedness |  |  |  |  |  |  |  | 1 | 1.860 | 0.173 |
|  | Dominance (3 levels) |  |  |  |  |  |  |  | 2 | 16.328 | **<0.001** |
|  | Relatedness:Dominance |  |  |  |  |  |  |  | 2 | 1.383 | 0.501 |
|  |  |  |  |  |  |  |  |  |  |  |  |
| (ii) Female response |  |  |  |  |  |  |  |  |  |  |  |
| (a) Proportion of mating attempts resisted | Relatedness |  |  |  |  | Results in Table 1 | | | 1 | 3.998 | **0.046** |
|  | Dominance (3 levels) |  |  |  |  |  |  |  | 2 | 0.631 | 0.729 |
|  | Relatedness:Dominance |  |  |  |  |  |  |  | 2 | 1.145 | 0.564 |
|  |  |  |  |  |  |  |  |  |  |  |  |
| (b) Average female resistance | Relatedness |  |  |  |  |  |  |  | 1 | 4.575 | **0.032** |
|  | Dominance (3 levels) |  |  |  |  |  |  |  | 2 | 1.166 | 0.558 |
|  | Relatedness:Dominance |  |  |  |  |  |  |  | 2 | 0.667 | 0.414 |
|  |  |  |  |  |  |  |  |  |  |  |  |
| (c) Probability of solicitation | Relatedness |  |  |  |  |  |  |  | 1 | 6.196 | **0.013** |
|  | Dominance (3 levels) |  |  |  |  |  |  |  | 2 | 0.629 | 0.730 |
|  | Relatedness:Dominance |  |  |  |  |  |  |  | 2 | 1.974 | 0.373 |
|  |  |  |  |  |  |  |  |  |  |  |  |
| (iii) Proportion of successful mating attempts |  |  |  |  |  |  |  |  |  |  |  |
|  | Relatedness |  |  |  |  | Results in Table 1 | | | 1 | 5.139 | **0.023** |
|  | Dominance (3 levels) |  |  |  |  |  |  |  | 2 | 1.822 | **0.610** |
|  | Relatedness:Dominance |  |  |  |  |  |  |  | 2 | 1.627 | 0.202 |
|  |  |  |  |  |  |  |  |  |  |  |  |
|  |  |  |  |  |  |  |  |  |  |  |  |
| POSTCOPULATORY RESPONSE |  |  |  |  |  |  |  |  |  |  |  |
|  |  | Dominance coded as Higher *vs.* Lower | | | |  | Dominance coded as 1,2,3 | | | | |
|  |  | Nested | Crossed | | | Nested | | | Crossed | | |
|  |  | Chi-sq | *df* | Chi-sq | *p* |  | Chi-sq | *p* |  | Chi-sq | *p* |
| (ii) Female response |  |  |  |  |  |  |  |  |  |  |  |
| (a) Probability of sperm ejection | Relatedness | Results in Table 2 | 1 | 0.670 | 0.413 |  |  |  |  |  |  |
|  | Relative dominance  (2 levels) |  | 1 | 0.784 | 0.376 |  |  |  |  |  |  |
|  | Relatedness:Relative dominance |  | 1 | 0.018 | 0.892 |  |  |  |  |  |  |
|  |  |  |  |  |  |  |  |  |  |  |  |
| (c) Number of hydrolysis points | Relatedness |  | 1 | 2.735 | 0.098 |  |  |  |  |  |  |
|  | Relative dominance  (2 levels) |  | 1 | 0.374 | 0.541 |  |  |  |  |  |  |
|  | Relatedness:Relative dominance |  | 1 | 0.849 | 0.357 |  |  |  |  |  |  |
|  | Relatedness:Lay date |  | 1 | 4.791 | **0.029** |  |  |  |  |  |  |

**Table S3**. Number of trials in which the single unrelated male exhibited a particular status.

| Precopulatory trials | |
| --- | --- |
| Dominance rank | Number of trials in which the unrelated male exhibited this status |
| 1 | 9 |
| 2 | 11 |
| 3 | 8 |
|  | X^2^ = 0.5, df = 2, p = 0.779 |
|  |  |
| Postcopulatory trials (sperm allocation experiments) | |
| Dominance rank | Number of trials in which the unrelated male exhibited this status |
| 1 | 14 |
| 2 | 9 |
| 3 | 8 |
|  | X^2^ = 2.0, df = 2, p = 0.368 |
|  |  |
| Postcopulatory trials (cryptic female choice) | |
| Dominance rank | Number of trials in which the unrelated male (focal male) exhibited this status |
| 1 | 11 |
| 2 | 5 |
| 3 | 6 |
|  | X^2^ =2.82, df = 2, p = 0.244 |
|  |  |
| Dominance rank | Number of trials in which the unrelated male (non-focal male) exhibited this rank |
| 1 | 5 |
| 2 | 5 |
| 3 | 12 |
|  | X^2^ = 4.5, df = 2, p = 0.108 |

**Table S4**. Untransformed estimates of fixed effects and covariates. Error structures are stated in brackets at the end of response variables. (A) Precopulatory experiments. (B) Postcopulatory experiments. Letters at the end of fixed effects indicate the level: U – unrelated, L – lower, Y – young. Female response of proportion of sperm ejected is not shown as there were only 7 samples where sperm was ejected and we used a Mann-Whitney U test.

A

|  | **Estimate** | **Std. Error** |
| --- | --- | --- |
| (i) Male behaviour | | |
| (a) Proportion of mating attempts interrupted (binomial) | | |
| (Intercept) | -2.040 | 0.377 |
| RelatednessU | 0.469 | 0.345 |
| Dominance DifferenceL | -0.659 | 0.435 |
| Year2011 | 0.270 | 0.313 |
| RelatednessU:Dominance DifferenceL | 0.168 | 0.498 |
|  |  |  |
| (b) Number of aggressive events (poisson) | | |
| (Intercept) | 0.034 | 0.333 |
| RelatednessU | 0.543 | 0.361 |
| Dominance DifferenceL | -3.500 | 0.686 |
| RelatednessU:Dominance DifferenceL | 0.033 | 0.739 |
|  |  |  |
| (c) Aggression level of interruption (cumulative) | | |
| RelatednessU | -0.108 | 0.436 |
| Dominance DifferenceL | -0.138 | 0.496 |
| RelatednessU:Dominance DifferenceL | -0.023 | 0.578 |
|  |  |  |
| (d) Courtship counts before attempt (poisson) | | |
| (Intercept) | 0.518 | 0.310 |
| Relatedness of FocalU | -0.243 | 0.489 |
| Focal Dominance2 | -1.405 | 0.458 |
| Focal Dominance3 | -0.729 | 0.423 |
| Relatedness of FocalU:Focal Dominance2 | 1.316 | 0.795 |
| Relatedness of FocalU:Focal Dominance3 | 0.994 | 0.821 |
|  |  |  |
| (e) Number of male-initiated attempts (poisson) | | |
| (Intercept) | 0.507 | 0.199 |
| Relatedness of FocalU | 0.061 | 0.288 |
| Focal Dominance2 | -0.385 | 0.238 |
| Focal Dominance3 | -0.577 | 0.233 |
| Year2011 | 0.506 | 0.195 |
| Relatedness of FocalU:Focal Dominance2 | 0.322 | 0.421 |
| Relatedness of FocalU:Focal Dominance3 | -0.370 | 0.461 |
| (ii) Female response | | |
| (a) Proportion of mating attempts resisted (binomial) | | |
| (Intercept) | 0.749 | 0.491 |
| Relatedness of FocalU | -0.688 | 0.444 |
| Focal Dominance2 | -0.197 | 0.338 |
| Focal Dominance3 | -0.179 | 0.341 |
| Year2011 | 0.184 | 0.333 |
| Relatedness of FocalU:Focal Dominance2 | -0.074 | 0.706 |
| Relatedness of FocalU:Focal Dominance3 | 0.555 | 0.772 |
|  |  |  |
| (b) Average female resistance (cumulative) | | |
| Relatedness of FocalU | -0.606 | 0.213 |
| Focal Dominance2 | -0.236 | 0.165 |
| Focal Dominance3 | -0.162 | 0.164 |
| Year2011 | 0.146 | 0.168 |
| Relatedness of FocalU:Focal Dominance2 | 0.227 | 0.343 |
| Relatedness of FocalU:Focal Dominance3 | 0.465 | 0.374 |
|  |  |  |
| (c) Probability of solicitation (binomial) | | |
| (Intercept) | -22.448 | 505.721 |
| Relatedness of FocalU | 19.915 | 505.719 |
| Focal Dominance2 | 19.187 | 505.719 |
| Focal Dominance3 | 0.164 | 538.378 |
| Year2011 | -0.302 | 0.690 |
| Relatedness of FocalU:Focal Dominance2 | -18.592 | 505.719 |
| Relatedness of FocalU:Focal Dominance3 | 0.819 | 538.378 |
|  |  |  |
| (a) Mating success (binomial) | | |
| (Intercept) | -1.052 | 0.511 |
| Relatedness of FocalU | 0.935 | 0.507 |
| Focal Dominance2 | 0.001 | 0.392 |
| Focal Dominance3 | -0.058 | 0.414 |
| Year2011 | -0.884 | 0.295 |
| Relatedness of FocalU:Focal Dominance2 | -0.276 | 0.805 |
| Relatedness of FocalU:Focal Dominance3 | -1.109 | 0.903 |

B

|  | **Estimate** | **Std. Error** |
| --- | --- | --- |
| (i) Male response | | |
| (a) Probability of investing sperm (binomial) | | |
| (Intercept) | -0.171 | 0.846 |
| RelatednessU | -0.161 | 1.016 |
| Dominance DifferenceL | 0.912 | 1.057 |
| Female AgeY | 18.911 | 4819.871 |
| Order2 | 1.184 | 0.657 |
| RelatednessU:Dominance DifferenceL | -0.975 | 1.471 |
|  |  |  |
| (b) Amount of sperm invested in 10^8^ counts of sperm (with aspermic attempts) | | |
| (Intercept) | 90.475 | 11.555 |
| RelatednessU | -15.524 | 12.326 |
| Dominance DifferenceL | 9.316 | 12.373 |
| Female AgeY | 14.694 | 11.371 |
| Order2 | 15.490 | 7.666 |
| RelatednessU:Dominance DifferenceL | -4.455 | 16.622 |
|  |  |  |
| (c) Amount of sperm invested in 10^8^ counts of sperm (without aspermic attempts) | | |
| (Intercept) | 86.127 | 15.324 |
| RelatednessU | -3.678 | 14.076 |
| Dominance DifferenceL | 18.356 | 15.336 |
| Female AgeY | 9.930 | 10.597 |
| Order2 | 21.381 | 8.272 |
| RelatednessU:Dominance DifferenceL | -20.227 | 18.775 |
|  |  |  |
| (ii) Female response | | |
| (a) Probability of sperm ejection (binomial) | | |
| (Intercept) | -1.129 | 1.218 |
| RelatednessU | -0.583 | 0.900 |
| Female AgeY | -0.013 | 0.928 |
| Number of sperm invested | -0.001 | 0.012 |
|  |  |  |
| (c) Number of hydrolysis points (poisson) |  |  |
| (Intercept) | 0.926 | 0.603 |
| RelatednessU | -0.539 | 0.634 |
| Lay Day | -0.182 | 0.068 |
| RelatednessU:Lay Day | 0.177 | 0.093 |
| DominanceL | -0.333 | 0.515 |
| Female AgeY | 0.608 | 0.481 |
| Number of sperm invested | -0.010 | 0.013 |

**Table S5**. Effects of random factors and covariates. (A) Precopulatory experiments. (B) Postcopulatory experiments. E-08 indicate 10^-8^ etc. Female response of proportion of sperm ejected is not shown as there were only 7 samples where sperm was ejected and we used a Mann-Whitney U test.

**A**

| **Response variable** | **Factors** | **Type** | **Values (mean ± S.E. for covariates, S.D. for random factors)** | **df** | **Test statistic** | ***P*** |
| --- | --- | --- | --- | --- | --- | --- |
| (i) Male behaviour | | | | | | |
| (a) Proportion of mating attempts interrupted | Year | Fixed | 2010: 0.154 ± 0.019; 2011: 0.180 ± 0.014 | 1 | 0.62 | 0.432 |
|  | Female:(Attempting:(Interrupting:Trial)) | Random | 5.40E-09 |  |  |  |
|  | Attempting:(Interrupting:Trial) | Random | 1.46E-08 |  |  |  |
|  | Interrupting:Trial | Random | 0.970 |  |  |  |
|  | Trial | Random | 0.221 |  |  |  |
|  |  |  |  |  |  |  |
| (b) Number of aggressive events | Observation level | Random | 1.027 |  |  |  |
|  | Aggressor:(Recipient:Trial) | Random | 0.535 |  |  |  |
|  | Recipient:Trial | Random | 0.978 |  |  |  |
|  | Trial | Random | 0.000 |  |  |  |
|  |  |  |  |  |  |  |
| (c) Aggression level of interruption | Attempting:(Interrupting:Trial) | Random | 3.79E-08 |  |  |  |
|  | Interrupting:Trial | Random | 0.299 |  |  |  |
|  | Trial | Random | 0.374 |  |  |  |
|  |  |  |  |  |  |  |
| (d) Courtship counts before attempt | Female:(Attempting:Trial) | Random | 2.15E-08 |  |  |  |
|  | Observation level | Random | 1.216 |  |  |  |
|  | Attempting:Trial | Random | 0.000 |  |  |  |
|  | Trial | Random | 0.411 |  |  |  |
|  |  |  |  |  |  |  |
| (e) Number of male-initiated attempts | Year | Fixed | 2010: 1.620 ± 0.175; 2011: 2.703 ± 0.215 | 1 | 5.98 | **0.015** |
|  | Female:(Attempting:Trial) | Random | 0.387 |  |  |  |
|  | Observation level | Random | 0.000 |  |  |  |
|  | Attempting:Trial | Random | 0.484 |  |  |  |
|  | Trial | Random | 0.293 |  |  |  |
|  |  |  |  |  |  |  |
| (ii) Female response | | | | | | |
| (a) Proportion of mating attempts resisted | Year | Fixed | 2010: 0.554± 0.038; 2011: 0.602 ± 0.025 | 1 | 0.32 | 0.574 |
|  | Male:(Female:Trial) | Random | 0.000 |  |  |  |
|  | Female:Trial | Random | 0.688 |  |  |  |
|  | Trial | Random | 0.478 |  |  |  |
|  |  |  |  |  |  |  |
| (b) Average female resistance | Year | Fixed | 2010: 3.179 ± 0.086; 2011: 3.321 ± 0.058 | 1 | 0.76 | 0.383 |
|  | Male:(Female:Trial) | Random | 1.33E-05 |  |  |  |
|  | Female:Trial | Random | 0.388 |  |  |  |
|  | Trial | Random | 0.231 |  |  |  |
|  |  |  |  |  |  |  |
| (c) Probability of solicitation | Year | Fixed | 2010: 0.056 ± 0.022; 2011: 0.042 ± 0.017 | 1 | 0.07 | 0.795 |
|  | Male:(Female:Trial) | Random | 0.639 |  |  |  |
|  | Female:Trial | Random | 1.68E-07 |  |  |  |
|  | Trial | Random | 0.160 |  |  |  |
|  |  |  |  |  |  |  |
| (iii) Mating success | | | | | | |
| (a) Mating success | Year | Fixed | 2010: 0.512 ± 0.083; 2011: 0.244 ± 0.0365 | 1 | 7.81 | **0.005** |
|  | Observation level | Random | 0.194 |  |  |  |
|  | Female:(Male:Trial) | Random | 0.000 |  |  |  |
|  | Male:Trial | Random | 0.000 |  |  |  |
|  | Trial | Random | 0.453 |  |  |  |
|  |  |  |  |  |  |  |

**B**

| **Response variable** | **Factors** | **Values (mean ± S.E.)** | **Values (mean ± S.E. for covariates, S.D. for random factors)** | **df** | **Test statistic** | ***p*** | |
| --- | --- | --- | --- | --- | --- | --- | --- |
| (i) Male response | | | | | | |  |
| (a) Probability of investing sperm | Female age | Fixed | young: 1.00 ± 0.00; old: 0.580 ± 0.071 | 1 | 8.31 | 0.004 | |
|  | Treatment order | Covariate | 1st: 0.58 ± 0.09;  2nd: 0.74 ± 0.08 | 1 | 2.65 | 0.104 | |
|  | Trial | Random | 0.954 |  |  |  | |
|  | Focal male identity | Random | 0.871 |  |  |  | |
|  |  |  |  |  |  |  | |
| (b) Amount of sperm invested | Female age | Fixed | young: 2.22 ± 0.49; old: 1.54 ± 0.37 | 1 | 1.94 | 0.164 | |
| in 10^8^ counts of sperm | Treatment order | Covariate | 1st: 1.61 ± 0.53;  2nd: 1.84 ± 0.34 | 1 | 3.78 | 0.052 | |
| (with aspermic attempts) | Trial | Random | 12.940 |  |  |  | |
|  | Focal male identity | Random | 15.750 |  |  |  | |
|  | Residual | Random | 22.750 |  |  |  | |
|  |  |  |  |  |  |  | |
| (c) Amount of sperm invested | Female age | Fixed | young: 2.22 ± 0.49; old: 1.67 ± 0.51 | 1 | 1.15 | 0.283 | |
| in 10^8^ counts of sperm | Treatment order | Covariate | 1st: 1.63 ± 0.59;  2nd: 2.12 ± 0.44 | 1 | 5.61 | **0.018** | |
| (without aspermic attempts) | Trial | Random | 9.84E-07 |  |  |  | |
|  | Focal male identity | Random | 25.040 |  |  |  | |
|  | Residual | Random | 22.460 |  |  |  | |
|  |  |  |  |  |  |  | |
| (ii) Female response |  |  |  |  |  |  | |
| (a) Probability of sperm ejection | Female age | Fixed | young: 0.200 ± 0.107; old: 0.182 ± 0.084 | 1 | 0.00 | 0.987 | |
|  | Number of sperm invested | Covariate |  | 1 | 0.01 | 0.942 | |
|  | Male:Female | Random | 0.000 |  |  |  | |
|  | Female | Random | 0.444 |  |  |  | |
|  |  |  |  |  |  |  | |
| (c) Number of hydrolysis points | Female age | Fixed | young: 7.08 ± 2.63; old: 2.49 ± 0.39 | 1 | 1.12 | 0.290 | |
|  | Number of sperm invested | Covariate |  | 1 | 0.58 | 0.450 | |
|  | Observation level | Random | 1.018 |  |  |  | |
|  | Male:Female | Random | 0.898 |  |  |  | |
|  | Female | Random | 0.155 |  |  |  | |

**Table S6**. Means and SE of each level of relatedness and dominance combination. (A) Precopulatory experiments. (B) Postcopulatory experiments. Columns are separated by dominance status. Female response of proportion of sperm ejected is not shown as there were only 7 samples where sperm was ejected.

**A**

| (i) Male behaviour | | | | | |
| --- | --- | --- | --- | --- | --- |
| (a) Proportion of mating attempts interrupted | | | | | |
|  | Higher | Lower |  | |  |
| Related | 0.187 ± 0.032 | 0.105 ± 0.021 |  | |  |
| Unrelated | 0.242 ± 0.025 | 0.153 ± 0.017 |  | |  |
|  |  |  |  | |  |
| (b) Number of aggressive events | | |  | |  |
|  | Higher | Lower |  | |  |
| Related | 2.152 ± 0.432 | 0.200 ± 0.129 |  | |  |
| Unrelated | 3.213 ± 0.498 | 0.289 ± 0.133 |  | |  |
|  |  |  |  | |  |
| (c) Aggression level of interruption | | |  | |  |
|  | Higher | Lower |  | |  |
| Related | 1.526 ± 0.160 | 1.588 ± 0.173 |  | |  |
| Unrelated | 1.712 ± 0.092 | 1.604 ± 0.082 |  | |  |
|  |  |  |  | |  |
| (d) Courtship counts before attempt | | | | | |
|  | 1 | 2 | 3 | |  |
| Related | 2.867 ± 1.591 | 0.417 ± 0.336 | 3.235 ± 1.413 | |  |
| Unrelated | 3.758 ± 0.609 | 1.750 ± 0.435 | 1.323 ± 0.295 | |  |
|  |  |  |  | |  |
| (e) Number of male-initiated attempts (poisson) | | | | | |
|  | 1 | 2 | 3 |  |  |
| Related | 2.657 ± 0.393 | 1.375 ± 0.261 | 1.683 ± 0.365 |  |  |
| Unrelated | 3.184 ± 0.446 | 2.492 ± 0.263 | 1.488 ± 0.259 |  |  |
|  |  |  |  |  |  |
| (ii) Female response | | | | | |
| (a) Proportion of mating attempts resisted | | | | | |
|  | 1 | 2 | 3 | |  |
| Related | 0.631 ± 0.039 | 0.613 ± 0.048 | 0.596 ± 0.050 | |  |
| Unrelated | 0.571 ± 0.050 | 0.479 ± 0.059 | 0.548 ± 0.091 | |  |
|  |  |  |  | |  |
| (b) Average female resistance |  |  |  | |  |
|  | 1 | 2 | 3 | |  |
| Related | 3.439 ± 0.088 | 3.340 ± 0.109 | 3.354 ± 0.115 | |  |
| Unrelated | 3.133 ± 0.124 | 3.000 ± 0.126 | 3.129 ± 0.235 | |  |
|  |  |  |  | |  |
| (c) Probability of solicitation | | | | | |
|  | 1 | 2 | 3 | |  |
| Related | 0.000 ± 0.000 | 0.039 ± 0.027 | 0.000 ± 0.000 | |  |
| Unrelated | 0.074 ± 0.051 | 0.121 ± 0.058 | 0.167 ± 0.078 | |  |
|  |  |  |  | |  |
| (a) Mating success | | | | | |
|  | 1 | 2 | 3 | |  |
| Related | 0.32 ± 0.083 | 0.283 ± 0.078 | 0.212 ± 0.068 | |  |
| Unrelated | 0.671 ± 0.147 | 0.465 ± 0.107 | 0.266 ± 0.111 | |  |

**B**

| (i) Male response | | |
| --- | --- | --- |
| (a) Probability of investing sperm | | |
|  | Higher | Lower |
| Related | 0.643 ± 0.133 | 0.765 ± 0.106 |
| Unrelated | 0.615 ± 0.140 | 0.611 ± 0.118 |
|  |  |  |
| (b) Amount of sperm invested in 10^8^ counts of sperm (with aspermic attempts) | | |
|  | Higher | Lower |
| Related | 1.584 ± 0.392 | 0.252 ± 0.683 |
| Unrelated | 0.902 ± 0.430 | 1.565 ± 0.585 |
|  | | |
| (c) Amount of sperm invested in 10^8^ counts of sperm (without aspermic attempts) | | |
|  | Higher | Lower |
| Related | 1.246 ± 0.467 | 2.815 ± 0.779 |
| Unrelated | 1.020 ± 0.477 | 1.744 ± 0.704 |
|  |  |  |
| (ii) Female response | | |
| (a) Probability of sperm ejection | | |
|  | Higher | Lower |
| Related | 0.231 ± 0.122 | 0.250 ± 0.250 |
| Unrelated | 0.091 ± 0.091 | 0.222 ± 0.147 |
|  |  |  |
| (c) Number of hydrolysis points | | |
|  | Higher | Lower |
| Related | 3.930 ± 0.967 | 2.818 ± 2.040 |
| Unrelated | 10.080 ± 6.042 | 2.596 ± 0.515 |
|  |  |  |
