# Supplementary material for: The contrasting role of male relatedness in different mechanisms of sexual selection in red junglefowl
Source: Evolution. 2017 Jan 5;71(2):403–20. doi: 10.1111/evo.13145 (PMC5324671; doi:10.1111/evo.13145)
Supplement: Supplementary file 2 — Figure S1. Male hierarchy on day 0 and during experiment. [file EVO-71-403-s002.docx]

Figure S1. Male hierarchy on day 0 and during experiment. (A). Relationship between avoidance counts before trials and average avoidance counts during trials. The relationship was highly significant (χ^2^_1_ = 64.24, p < 0.001) when analysed using a General linear mixed model with ‘year’ and ‘relative dominance different’ as fixed factors, ‘aggressor identity’ nested within ’recipient identity’ as a random factor. (B) Average number of times each male of different dominance status was avoided before trials (day 0) and during trials.
