# Supplementary material for: The contrasting role of male relatedness in different mechanisms of sexual selection in red junglefowl
Source: Evolution. 2017 Jan 5;71(2):403–20. doi: 10.1111/evo.13145 (PMC5324671; doi:10.1111/evo.13145)
Supplement: Supplementary file 3 — Figure S2. Repeatability of male aggression behaviour across trials. [file EVO-71-403-s003.docx]

Figure S2. Repeatability of male aggression behaviour across trials. We tested the correlations using a General linear mixed model with ‘relatedness’ as a fixed factor, ‘aggressor identity’ nested within ’recipient identity’ as a random factor and observation-level random factor to account for overdispersion. The correlations were highly significant: 3^rd^ period against 1^st^ (χ^2^_1_ = 13.81, p < 0.001); 3^rd^ period against 2^nd^ (χ^2^_1_ = 13.85, p < 0.001); 2^nd^ period against 1^st^ (χ^2^_1_ = 13.21, p < 0.001).

Aggression levels during 1^st^ period

Aggression levels during 2^nd^ period

Aggression levels during 3^rd^ period

SIMPLE CORRELATION
